# Myasthenia Gravis: Analysis of Serum Autoantibody Reactivities to 1827 Potential Human Autoantigens by Protein Macroarrays

**DOI:** 10.1371/journal.pone.0058095

**Published:** 2013-03-04

**Authors:** Anne Becker, Nicole Ludwig, Andreas Keller, Björn Tackenberg, Christian Eienbröker, Wolfgang H. Oertel, Klaus Fassbender, Eckart Meese, Klemens Ruprecht

**Affiliations:** 1 Department of Human Genetics, Universität des Saarlandes, Homburg, Germany; 2 Siemens Healthcare, Erlangen, Germany; 3 Clinical Neuroimmunology Group, Department of Neurology, Philipps-Universität Marburg, Marburg, Germany; 4 Department of Neurology, Universität des Saarlandes, Homburg, Germany; 5 Department of Neurology, Charité – Universitätsmedizin Berlin, Berlin, Germany; Hannover Medical School, Germany

## Abstract

**Background:**

Myasthenia gravis is a disorder of neuromuscular transmission associated with autoantibodies against the nicotinic acetylcholine receptor. We have previously developed a customized protein macroarray comprising 1827 potential human autoantigens, which permitted to discriminate sera of patients with different cancers from sera of healthy controls, but has not yet been evaluated in antibody-mediated autoimmune diseases.

**Objective:**

To determine whether autoantibody signatures obtained by protein macroarray separate sera of patients with myasthenia gravis from healthy controls.

**Methods:**

Sera of patients with acetylcholine receptor antibody-positive myasthenia gravis (n = 25) and healthy controls (n = 32) were analyzed by protein macroarrays comprising 1827 peptide clones.

**Results:**

Autoantibody signatures did not separate patients with myasthenia gravis from controls with sufficient sensitivity, specificity, and accuracy. Intensity values of one antigen (poly A binding protein cytoplasmic 1, p = 0.0045) were higher in patients with myasthenia gravis, but the relevance of this and two further antigens, 40S ribosomal protein S13 (20.8% vs. 0%, p = 0.011) and proteasome subunit alpha type 1 (25% vs. 3.1%, p = 0.035), which were detected more frequently by myasthenia gravis than by control sera, currently remains uncertain.

**Conclusion:**

Seroreactivity profiles of patients with myasthenia gravis detected by a customized protein macroarray did not allow discrimination from healthy controls, compatible with the notion that the autoantibody response in myasthenia gravis is highly focussed against the acetylcholine receptor.

## Introduction

Myasthenia gravis (MG) is an overall rare disorder of neuromuscular transmission, clinically characterized by fluctuating muscle weakness and abnormal fatigability [1]. Initially, weakness may be confined to extrinsic ocular muscles (ocular MG), but it frequently progresses to bulbar and limb muscles (generalized MG) [2]. Weakness is caused by T-helper cell dependent autoantibodies against the nicotinic acetylcholine receptor (AChR antibodies), which can be detected in approximately 80–90% of patients with generalized MG [1], [2], [3], [4]. In addition, serum autoantibodies against a number of different antigens have been reported in patients with MG [5]. Among these are antibodies against myosin [6], filamin, vinculin, and tropomyosin [7], as well as rapsyn [8]. Further non-AChR antibodies have been described especially in thymoma-associated or late-onset MG, including antibodies against α-actinin and actin [9], [10], and neutralizing antibodies against interferon (IFN)-α, IFN-ω, and interleukin (IL)-12 [11], [12], [13]. Patients with thymoma-associated MG often also have antibodies against the striational muscle proteins titin and ryanodine receptor [14], [15], [16], [17]. Although non-AChR autoantibodies are generally less frequently detectable than AChR antibodies, the existence of such non-AChR autoantibodies opens the possibility of a more globally disturbed serum autoantibody profile in patients with MG.

Protein macroarrays are a tool for simultaneous detection of multiple autoantibody reactivities [18], [19]. Evaluation of autoantibody profiles from protein macroarrays is based on the assumption that analysis of patterns of multiple antibody reactivities might be more informative than analysis of single autoantibodies alone [20]. Indeed, previous work performed in patients with various cancers as well as autoimmune diseases suggests that autoantibody profiles may have the potential to serve as disease biomarkers and to provide clues for disease pathogenesis [20], [21], [22], [23], [24], [25], [26]. Accordingly, a customized protein macroarray comprising 1827 potential human autoantigens recently developed in our laboratory permitted to adequately discriminate sera of patients with different cancers from sera of healthy controls [27], [28], [29]. However, this customized macroarray has not yet been evaluated in antibody-mediated autoimmune diseases.

Taking MG as a prototypical model for an antibody-mediated autoimmune disease, we here analyzed autoantibody signatures in sera from patients with generalized MG and healthy controls by protein macroarrays comprising 1827 potential human autoantigens. The aims of this study were (i) to determine whether seroreactivity profiles obtained by protein macroarray permit serological discrimination of patients with MG from healthy controls and (ii) to identify novel antigenic targets of non-AChR autoantibodies in MG. Altogether, autoantibody profiles did not discriminate patients with MG from healthy controls with acceptable sensitivity, specificity, and accuracy, compatible with the notion that the autoantibody response in myasthenia gravis is highly focussed against the acetylcholine receptor.

## Patients and Methods

### Patients with myasthenia gravis and healthy controls

Sera from n = 25 patients (17 female, 8 male) with generalized AChR antibody-positive MG were collected by peripheral venipuncture at the Department of Neurology, Philipps-Universität Marburg, with approval of the institutional review board of the medical faculty of Philipps-Universität Marburg and written informed consent. Median (range) age of patients was 35 (16–86) years. Defining early- and late-onset MG as onset of the disease before and after the age of 50 years [30], there were 16 patients with early-onset MG (EOMG) and 9 patients with late-onset MG (LOMG). Diagnosis of MG was based on clinical presentation, presence of AChR antibodies, and electrophysiological findings. At the time of study entry 14 out of 25 (56%) patients with MG were treated with pyridostigmine. None of the patients with MG took glucocorticosteroids or any other immunosuppressive medications before or by the time of blood withdrawal. However, two of the 25 MG patients had undergone thymectomy prior to blood sampling. Another 14 patients were thymectomized at some point in time after blood collection. Histology results were available from 11 of 16 thymectomized patients showing normal thymus tissue in 1, thymic atrophy in 2, thymoma in 1, and thymic hyperplasia in 7 patients. All patients were also tested for antibodies to titin. 8/9 (88.9%) of patients with LOMG and 4/16 (25%) patients with EOMG were titin antibody-positive. The patient with thymoma-associated MG (age at onset: 16 years) had antibodies to titin. Concomitant autoimmune diseases were present in 4 patients (autoimmune thyroid disease, n = 3; antiphospholipid syndrome, n = 1). Control sera from healthy blood donors (n = 32; 21 female, 11 male) were obtained from the Department of Hemostaseology and Transfusion Medicine, Universität des Saarlandes, after written informed consent. Median (range) age of controls was 41.5 (19–64) years. Age (p = 0.8, unpaired *t* test) and gender distribution (p = 1, Fisher's exact test) did not significantly differ between the patient and control group. Patient and control sera were stored at –20°C.

### Protein macroarray screening

Protein macroarray screening was carried out as previously described [21], [27], [28]. In brief, a high-density protein macroarray containing 38,016 *E.coli* expressed peptide clones from the hex1 library [31] had been pre-screened with 150 sera from patients with various conditions, including cancer and autoimmune diseases. Peptide clones that bound (auto-)antibodies present in at least one of those sera were considered potential human autoantigens and assembled on a customized human autoantigen macroarray, which contained, in total, 1827 *E.coli* expressed clones, each spotted in duplicates on filter membranes. Customized macroarrays were produced by and obtained from ImaGenes, Berlin, Germany. For screening of sera from patients with MG and healthy controls, array membranes were incubated in 96% ethanol and rinsed two times with distilled water. After two washes with TBST-T (TBS, 0.05% Tween 20, 0.5% Triton X-100) and two washes with TBS, membranes were blocked in blocking solution (3% non-fat dry milk powder in TBST [TBS, 0.05% Tween 20]) for two hours. Subsequently, membranes were incubated overnight at 4°C with sera diluted 1∶1000 in blocking solution. After incubation, sera were collected and stored at 4°C for a second incubation round. Membranes were washed three times with TBST and incubated with heated stripping solution (70°C) for 30 minutes. After two washes with TBST and two washes with TBS membranes were again blocked with blocking solution for two hours followed by overnight incubation at 4°C with previously stored sera. Macroarrays were washed three times with TBST and incubated for two hours with a secondary rabbit anti-human IgG, IgM, IgA (H+L) Cy5-labelled antibody (Dianova, Hamburg, Germany) diluted 1∶1000 in blocking solution. Next, membranes were washed four times in TBST and twice in TBS, and dried overnight. Protein macroarrays were then scanned with a GE Healthcare Typhoon 9410 scanner at 570 nm and a resolution of 50 µm.

### Image analysis

Standardized evaluation of the scanned images was carried out by a previously described computer aided image analysis procedure [27], [28]. In pre-processing steps images were adjusted and edges virtually cut. Arrays were then segmented into subgrids that were further divided into target areas containing exactly one protein spot. By k-means clustering, pixels belonging to the spot area were divided into dark foreground and pale background pixels. For the extraction of dark protein spots, a morphological operator from image processing, the so-called black top hat operator, was applied to the image. The intensity of each spot was calculated as the mean value of the dark foreground pixels. Intensity values ranged from 0 to 255, that is, the standard range of values in a grey scale image. As each clone is represented in duplicates on the array, the mean of the intensity values of both replicates was assigned to each clone. In case no spot could be detected in the target area by the automated system, the respective peptide clone was marked as not available.

### Bioinformatical and statistical data analysis

To minimize interarray variation, intensity values of the 1827 peptide clones were first normalized using standard quantile normalization. To analyze whether autoantibody profiles determined by protein macroarrays can differentiate patients with MG from healthy controls we used standard linear kernel support vector machines with a 10-fold cross validation. The classification procedure was repeated 100 times and the mean sensitivity, specificity, and accuracy were calculated together with 95% confidence intervals. As a test for overfitting, the entire classification procedure was also performed with randomly permutated class labels. We performed the same classification procedure also for EOMG vs. LOMG patients.

To directly determine the capacity of a given peptide clone to separate patients with MG from healthy controls, we calculated the area under the receiver operator characteristics curve (AUC) for each antigen. The receiver operator characteristics curve displays sensitivity as a function of one minus specificity. For calculation of AUC values, for each clone the normalized intensities of all analyzed sera were used as threshold values to differentiate sera from patients with MG from healthy controls. For all thresholds *t* we considered sera from patients with MG with an intensity value above *t* as true positives (TP) and sera with intensity values below *t* as false negatives (FN). Likewise, sera from healthy controls with intensity values above *t* were classified as false positives (FP) and with intensity values below *t* as true negatives (TN). For all thresholds, we computed sensitivity (TP/(TP + FN) and specificity (TN/TN + FP). AUC values can range between 0 and 1. An AUC of 0.5 indicates that the distribution of the intensity values from patients with MG and healthy controls cannot be distinguished. AUC values <0.5 indicate that intensity values in sera from patients with MG are higher than those from healthy controls, and AUC values >0.5 indicate that intensity values of sera from healthy controls are higher than those from patient with MG. Only AUC values <0.3 and >0.7 were considered informative in this analysis. We also plotted normalized intensity values of informative clones and assessed statistical significance of differences by two-tailed Mann-Whitney U test using GraphPad Prism 5.03.

The frequency of positive seroreactivities among the groups of patients with MG and healthy controls against a given peptide clone was calculated after arbitrarily defining intensity values ≥50 as positive and intensity values <50 as negative [28]. Statistical significance of different frequencies of serum autoantibodies was determined using two-tailed Fisher's exact probability test. Calculations were performed using VassarStats (http://faculty.vassar.edu/lowry/VassarStats.html) and p-values below 0.05 were considered significant.

For stochastic reasons, only one third of clones spotted on the macroarray are in the correct reading frame (in-frame) with respect to the start codon of the vector used for *E. coli* transformation [31]. Identity of the proteins encoded by those in-frame clones can be determined by sequencing of the inserted cDNAs. In contrast, about two thirds of clones are out-of-frame with respect to the vector start codon. While such out-of-frame clones may still produce the correct protein encoded by the inserted cDNA fragment, for instance, in case of a translational start within the cDNA insert, out-of-frame clones may also produce non-sense or truncated proteins resulting from translation in a wrong reading frame [31]. Although it is well documented that proteins produced by out-of-frame clones may readily be recognized by serum (auto)antibodies in protein macroarray experiments [22], [31], [32], for the reasons discussed above, the precise identity of those proteins is difficult to determine. We therefore focussed our analyses on in-frame clones.

## Results

### Serum autoantibody profiles do not discriminate between patients with MG and controls

**Figure 1:**
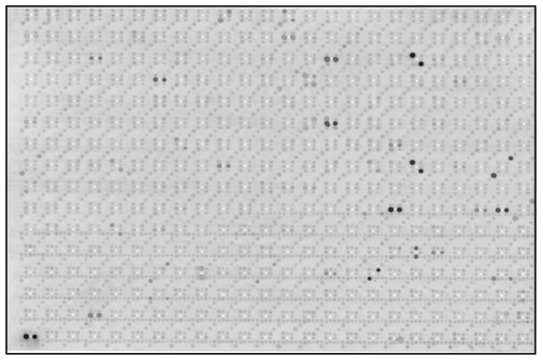
A customized protein macroarray containing 1827 potential human autoantigens was incubated with serum from a patient with MG and developed with a secondary Cy5-labelled anti human immunoglobulin antibody. Shown is a scanned image of the macroarray (GE Healthcare Typhoon 9410 scanner) as used for the subsequent automated image analysis procedure. Since peptide clones are spotted in duplicates on the filter membrane, positive seroreactivities are represented by two dark spots.

To characterize autoantibody profiles in patients with MG we screened sera from 25 patients with AChR antibody-positive generalized MG and 32 healthy controls with a customized protein macroarray containing 1827 potential human autoantigens. Scanned images from all arrays were evaluated with an automated image analysis procedure, yielding 1827 intensity values, ranging from 0 to 255, for each tested serum. After quantile normalization 102 peptide clones were excluded from further analyses as they were marked as not available in more than 10 sera by the image analysis program. Normalized intensity values of the remaining 1725 peptide clones were used for all subsequent calculations. An example of a scanned protein macroarray is shown in Figure 1.

To determine whether global serum autoantibody reactivities against the 1725 peptide clones separate patients with MG from healthy controls we applied linear kernel support vector machines with 100 repetitions of a 10-fold cross validation. As shown in Table 1, the mean sensitivity (43.4%), specificity (62%), and accuracy (53.9%) for the classification of MG versus healthy controls were rather moderate. Moreover, these values were not substantially different from the mean sensitivity, specificity, and accuracy obtained after random permutation of the class labels. Altogether, these data indicate that global autoantibody profiles, as determined by the employed set of potential human autoantigens, do not discriminate between patients with MG and healthy controls.

**Table 1:** Classification results for myasthenia gravis vs. healthy controls and EOMG vs. LOMG.

Classification	Sensitivity (%)	Specificity (%)	Accuracy (%)
Myasthenia vs. Controls	43.4 (40.3–46,5)	62 (59.4–64.6)	53.9 (51.9–55.8)
Random	37 (32.5–41.5)	54.7 (51.5–57.9)	46.9 (44.0–49.9)
EOMG vs. LOMG	46.1 (42.5–49.7)	74.7 (70.3–79.1)	64.4 (61.0–67.8)
Random	23.9 (18.4–29.4)	68.8 (63.3–74.2)	52.6 (47.6–57.6)

Mean values for sensitivity, specificity, and accuracy for classification of patients with myasthenia gravis (n = 25) vs. healthy controls (n = 32) and EOMG (n = 16) vs. LOMG (n = 9) were calculated by linear kernel support vector machines with 100 repetitions of a 10-fold cross validation. 95% confidence intervals are indicated in parentheses. As control, classifications were performed with randomly permutated class labels.

We also analyzed whether seroreactivity profiles could separate EOMG from LOMG patients. The classification of EOMG versus LOMG yielded a mean sensitivity of 46.1%, a mean specificity of 74.7%, and a mean accuracy of 64.4% (Table 1). The classification with randomly permutated class labels yielded a mean sensitivity of 23.9%, a mean specificity of 68.8%, and a mean accuracy of 52.6%. Altogether, although this classification was better than random guessing, these data do not suggest that EOMG and LOMG patients can be separated with sufficient sensitivity, specificity, and accuracy by our customized peptide autoantigen macroarray. Likewise, an individual analysis of the seroreactivity profile of the one thymoma patient as compared to all other MG (EOMG and LOMG) patients showed a correlation with the seroreactivity profiles of more than half of all other MG patients, indicating that this patient's seroreactivity profile was not particularly different from that of all other MG patients (Figure S1).

### Analysis of AUC Values

To analyze whether intensity values of individual peptide clones differ between patients with MG and controls we calculated AUC values for all 1725 peptide clones. As shown in Table 2, which lists the number of peptide clones in different AUC value ranges, in total, 7 out of 1725 (0.4%) clones had AUC values <0.3 or >0.7 and were therefore considered potentially informative.

**Table 2:** Distribution of clones in different AUC value intervals.

AUC value range	Myasthenia vs. Controls	
	No. all clones	No. in-frame clones
0.0–0.1	0	0
0.1–0.2	0	0
0.2–0.3	**5**	**1**
0.3–0.4	99	34
0.4–0.5	760	220
0.5–0.6	706	184
0.6–0.7	153	37
0.7–0.8	**2**	0
0.8–0.9	0	0
0.9–1.0	0	0

Clones with AUC values <0.3 or >0.7 were considered informative and are marked in boldface.

**Figure 2:**
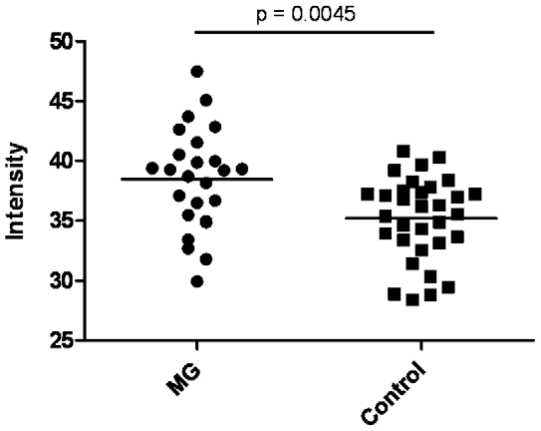
Normalized intensity values for poly A binding protein cytoplasmic 1 (PABPC1) in the groups of patients with MG and healthy controls. Horizontal bars represent median intensity values. Statistical significance of differences was assessed by Mann-Whitney U test.

Among the seven clones considered informative according to their AUC values there was one in-frame clone with sequence homology to poly A binding protein cytoplasmic 1 (PABPC1, Ensemble ID ENSG00000070756), which had an AUC value of 0.279. The absolute normalized intensity values for PABPC1 are depicted in Figure 2. Although intensity values of PABPC1 were significantly higher in patients with MG as compared to healthy controls (p = 0.0045, Mann-Whitney U test), there was a considerable overlap of the intensity values of both groups and absolute intensity values were rather low. In summary, calculation of AUC values for 1725 peptide clones revealed potentially informative differences of intensity values for only one in-frame clone with sequence homology to PABPC1.

### Frequent detection of a few peptide antigens by serum antibodies present in patients with MG and healthy controls

To determine the frequency with which particular peptide clones are recognized by antibodies present in sera from patients with MG vs. healthy controls we used an arbitrarily defined cut-off, considering intensity values of ≥50 as positive and <50 as negative [26], [28]. The frequency of positive sera for a given peptide clone in each group was then calculated according to the formula [(number of sera with intensity values ≥50/(number of sera with intensity values ≥50 + number of sera with intensity values <50)] ×100%. Table 3 shows the percentage of positive sera in the groups of patients with MG and healthy controls for different frequency ranges. In both groups, almost 800 peptide clones were completely negative, that is, those clones yielded intensity values <50 with the analyzed sera. Conversely, a few clones were recognized very frequently. More precisely, there were 4 peptides clones (including 1 in-frame clone; ubiquitin-fold modifier conjugating enzyme 1) with intensity values ≥50 in at least 80% of sera from patients with MG, and 8 peptide clones (including 1 in-frame clone; kinesin family member 18B) with intensity values ≥50 in at least 80% of sera from healthy controls. Closer analysis demonstrated that those frequently detected clones essentially overlapped between patients and controls. For example, antibodies against ubiquitin-fold modifier conjugating enzyme 1 were detected in 80% of MG sera and 71.9% of control sera and antibodies against kinesin family member 18B in 84.4% of control sera and 62.5% of MG sera.

**Table 3:** Distribution of clones in different seroreactivity frequency intervals.

Frequencies (%)	Myasthenia		Controls		Myasthenia ≥2× controls	
	No. all clones	No. in-frame clones	No. all clones	No. in-frame clones	No. all clones	No. in-frame clones
0	797	218	771	224	-	-
0-10	579	166	611	170	251	71
10-20	209	65	206	59	56	19
20-30	122	33	109	25	**17**	**5**
30-40	48	11	55	14	**6**	**2**
40-50	38	8	25	8	**1**	0
50-60	9	2	21	4	0	0
60-70	17	4	14	2	0	0
70-80	4	1	7	2	0	0
80-90	2	1	5	1	0	0
90-100	2	0	3	0	0	0

The number of clones reacting with antibodies in sera from patients with MG and healthy controls is listed according to the frequency of positive seroreactivities. The column „Myasthenia ≥2× controls” lists all clones that were positive at least twice as often with sera from patients with MG than with control sera. Among those, clones that were detected by at least 20% of MG sera were considered informative and are marked in boldface.

### Increased frequency of serum autoantibodies against RPS13 and PSMA1 in patients with MG

To determine whether any particular peptide clones are more frequently positive with sera from patients with MG compared to sera from healthy controls we subsequently identified all clones that were at least twice as often positive with sera from patients with MG compared to sera from healthy controls. In total, there were 331 peptide clones meeting this requirement (Table 3). To focus on potentially meaningful peptide clones we confined further analyses on clones that were positive in at least 20% of MG sera. This criterion applied to 24 clones, 7 of which were in-frame clones. We assessed the statistical significance of the differences in seropositivity rates of those 7 in-frame clones between the group of patients with MG and healthy controls by Fisher's exact probability test. Significant differences were observed for two clones, representing 40S ribosomal protein S13 (RPS13; p = 0.011) and proteasome subunit alpha type 1 (PSMA1; p = 0.035). Seropositivity rates for RPS13 and PSMA1 in patients with MG were 20.8% and 25%, whereas in the control group only 0% and 3.1% of sera were positive for these clones (Table 4).

**Table 4:** Details of peptide clones that were detected significantly more frequently by sera from patients with MG than by control sera.

Clone ID	Ensemble ID	Gene name	Frequency Myasthenia (%)	Frequency Controls (%)	p-value
K02549	ENSG00000110700	RPS13	5/24 (20.8)	0/32 (0)	0.011
E05529	ENSG00000129084	PSMA1	6/24 (25)	1/32 (3.1)	0.035

The number of positive sera (i.e. sera with intensity values >50; for details see text) out of all sera tested is indicated. P-values were determined by Fisher's exact probability test. RPS13, 40S ribosomal protein S13; PSMA1, proteasome subunit alpha type 1.

## Discussion

The main result of this study is that the autoantibody profiles obtained by the customized protein macroarray employed in this work do not permit to accurately differentiate patients with MG and healthy controls. This finding is in marked contrast to results from previous autoantibody screens carried out with the same autoantigen macroarray in patients with lung cancer, glioma, and meningioma [27], [28], [29]. Indeed, autoantibody signatures discriminated sera from patients with lung cancer from healthy control sera with a specificity, sensitivity, and accuracy of ≥97% each [27] and allowed to separate glioma sera from healthy control sera with a specificity of 90.5%, a sensitivity of 85.9%, and an accuracy of 88.5% [28]. Since in the current screen with sera from patients with MG we used exactly the same methodology as in the previous screens, different results are unlikely to be due to methodological issues. Intriguingly, global autoantibody profiles therefore appear to be more strongly perturbed in the investigated neoplastic diseases than in the prototypical antibody-mediated disease MG. A possible explanation for this finding is that the humoral immune response in patients with MG is highly focussed against the AChR, while the overall autoantibody repertoire remains largely unchanged. In contrast, the potential number of targets of an antitumoral immune response comprises the whole tumor proteome including mutated, misfolded, overexpressed, aberrantly degraded, or aberrantly glycosylated proteins [33]. The humoral immune response to tumor antigens may thus be much broader than the humoral autoimmune response in MG. Differentiation between the groups of EOMG and LOMG patients by the customized macroarray was similarly rather moderate, which would again be compatible with a highly focussed immune response against the AChR in EOMG as well as LOMG.

One important limitation of our study is that the employed protein macroarray is based on bacterially expressed peptide clones which produce protein fragments that are likely non-conformational and that do not undergo posttranslational modifications, such as glycosylation. Also, AChR peptides, or peptides derived from other antigens against which antibodies have been detected in MG, are not represented on the customized macroarray. AChR antibodies are usually specific for the native conformation of the AChR and rarely recognize peptide fragments efficiently [34]. Anti-AChR or antibodies against other conformational epitopes are therefore unlikely to be detected by the present approach. The failure to identify differences in the seroreactivity profiles of patients with MG vs. controls as well as EOMG vs. LOMG may therefore also be related to the absence of relevant autoantigens on the macroarray and the limited ability of peptide macroarrays to identify antibodies against conformational epitopes. Nevertheless, it should be emphasized that it was not the intention of this study to use protein macroarrays as an alternative method for the detection of known antibodies in patients with MG, but to evaluate whether patients with MG can be separated from controls based on seroreactivities against the peptide clones represented on the array. Given the large number of potential autoantigens assembled on the array, in case there was a globally changed autoantibody repertoire in MG, it seems likely that at least some of such autoantibody reactivities should be detectable by the protein macroarray approach.

While the exact length of the peptide clones on the array could not be determined, as the clones were only sequenced from the 5′ end, the minimum length of an open reading frame of an insert to be considered as an in-frame antigen was 30 amino acids. However, open reading frames of the peptide clones were generally several times longer (up to about 250 amino acids). It seems conceivable that there may be a qualitative difference in the humoral immune response to self-antigens of patients with MG and cancer, with antibodies from cancer patients having a higher propensity to recognize linear peptide epitopes of 30 to 250 amino acids while autoantibodies from patients with MG may be generally more likely to detect conformational epitopes. However, a more likely alternative explanation for the different results obtained with MG and cancer sera analyzed with the customized protein macroarray may be a quantitative difference in the immune response to autoantigens. Indeed, assuming that the autoantibody response in patients with cancer is much broader than in patients with MG, chances that some autoantibodies also interact with the linear peptide epitopes represented on the macroarray would be higher in patients with cancer than with MG.

Calculation of AUC values for 1725 peptide clones revealed one in-frame clone, with sequence homology to PABPC1, with an informative AUC value. PABPC1 is a cytoplasmic protein that specifically binds to poly A tails of mRNA and is involved in initiation of mRNA translation, mRNA stabilization, and regulation of mRNA decay [35]. PABPC1 immuno-positive deposits forming conglomerates with poly A RNA were previously found in muscle biopsies of patients with sporadic inclusion body myositis (IBM) leading to the speculation that an autoantibody-mediated inhibition of mRNA degradation could play a role in the pathogenesis of IBM [36]. The detection of antibodies against a peptide with homology to PABPC1 in human sera therefore appears interesting, however, the significance of these autoantibodies in patients with MG currently remains elusive.

Using an arbitrary cut-off of an intensity value >50 we found a few peptide clones which were very frequently detected by MG and healthy control sera. We speculate that these autoantibodies could represent natural autoantibodies, that are known to react with foreign but also self antigens [37]. Alternatively, those frequently detected peptide clones could represent mimotopes, that is, antigenic epitopes that are structurally similar, for instance, to epitopes recognized by antibodies against common viruses.

We identified two in-frame clones, RPS13 and PSMA1, that were detected significantly more often by sera from patients with MG than by healthy control sera. RPS13 is a cytoplasmic protein and part of the 40S ribosomal subunit, autoantibodies against which have previously been identified by a phage-display technique in patients with systemic lupus erythematosus (SLE) [38]. PSMA1 represents one of the seven α-subunits of the outer rings of the 20S-proteasome [39]. Antibodies against proteasomal subunits were previously described in patients with SLE, myositis, and Sjögren's syndrome [39]. A detailed analysis of the fine specificity of antibodies against the different proteasomal subunits demonstrated antibodies against PSMA1 (also termed C2) in sera of patients with undifferentiated connective tissue disease and Wegener's granulomatosis [39]. Antibodies against proteasomal subunits, including C2, have also been observed in multiple sclerosis [40]. The presence of antibodies against RPS13 and PSMA1 in different autoimmune diseases suggests that those autoantibodies are not specifically associated with MG, but could represent a phenomenon associated with autoimmunity in general. The clone representing PABPC1 covered amino acids (aa) 173-460 of PABPC1 (total length 637 aa), the RPS13 clone covered amino acids 13-151 of RPS1 (151 aa), and the clone representing PSMA1 covered the entire PSMA1 protein (263 aa). Still, our data do not formally prove that the antibodies reacting with these clones are indeed directed against PABPC1, RPS13, and PSMA1 protein. As discussed above, these proteins are likely presented as linearized peptides on the macroarray and could also function as mimotopes for antibodies to proteins other than PABPC1, RPS13, and PSMA1. Furthermore, our data await reproduction by a different method such as ELISA or western blot and the seropositivity rates for RPS13 (20.8%) and PSMA1 (25%) in MG were overall rather low, thus limiting their potential diagnostic value. Altogether, the overall relevance of PAPBC1, RPS13, and PSMA1 in MG appears uncertain and it would be premature to regard them as novel targets of non-AChR autoantibodies in patients with MG.

In conclusion, a customized protein macroarray comprising 1827 immunogenic peptide clones could not discriminate sera of patients with generalized MG and sera of healthy controls with sufficient sensitivity, specificity, and accuracy. We identified one clone (PABPC1) with an informative AUC value for the separation of MG from healthy controls and two clones, RPS13 and PSMA1 which were more frequently detected by sera from patients with MG than by healthy control sera. The relevance of antibodies to these antigens in MG is currently unknown and it remains uncertain whether those antigens may represent novel antigenic targets in MG. Given previous data on the successful serological differentiation of patients with various forms of cancer from healthy controls by the protein macroarray used in this work, the absence of overt distortions of global autoantibody signatures in MG appears remarkable and suggests that the humoral immune response in patients with MG is highly focussed against the AChR.

## Supporting Information

Figure S1 (PPT)
