# Supplementary material for: Myasthenia Gravis: Analysis of Serum Autoantibody Reactivities to 1827 Potential Human Autoantigens by Protein Macroarrays
Source: PLoS One. 2013 Mar 4;8(3):e58095. doi: 10.1371/journal.pone.0058095 (PMC3587426; doi:10.1371/journal.pone.0058095)
Supplement: Figure S1 — (PPT) [file pone.0058095.s001.ppt]

## Slide 1
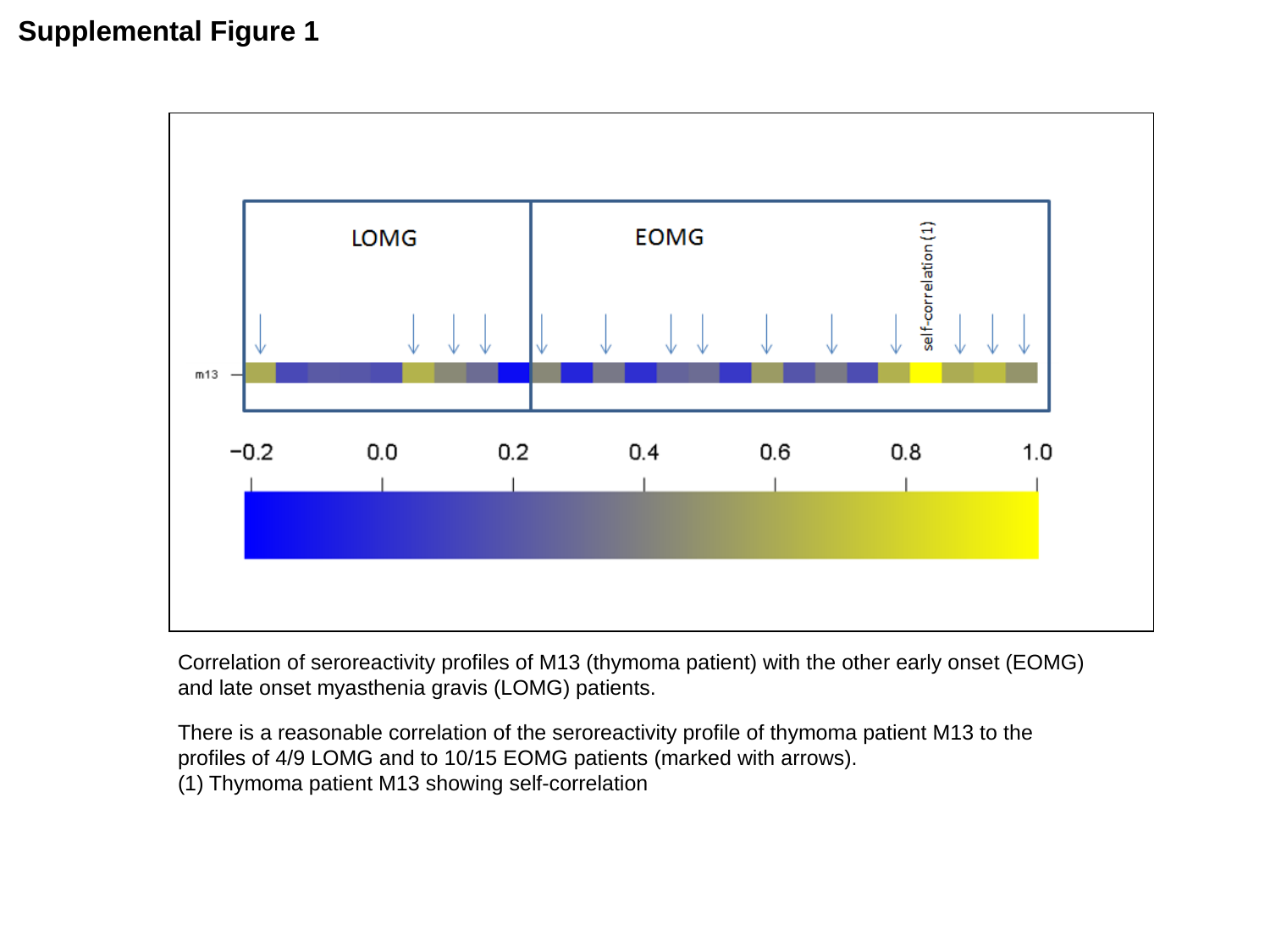

Supplemental Figure 1
Correlation of seroreactivity profiles of M13 (thymoma patient) with the other early onset (EOMG) and late onset myasthenia gravis (LOMG) patients.
There is a reasonable correlation of the seroreactivity profile of thymoma patient M13 to the profiles of 4/9 LOMG and to 10/15 EOMG patients (marked with arrows).
(1) Thymoma patient M13 showing self-correlation
